# Deep Proteomics Analysis Unravels the Molecular Signatures of Tonsillar B Cells in PFAPA and OSAS in the Pediatric Population

**DOI:** 10.3390/ijms26146621

**Published:** 2025-07-10

**Authors:** Feras Kharrat, Nour Balasan, Blendi Ura, Valentina Golino, Pietro Campiglia, Giulia Peri, Erica Valencic, Mohammed Qaisiya, Ronald de Moura, Mariateresa Di Stazio, Barbara Bortot, Alberto Tommasini, Adamo Pio d’Adamo, Egidio Barbi, Domenico Leonardo Grasso

**Affiliations:** 1 Institute for Maternal and Child Health, IRCCS Burlo Garofolo, 65/1 Via dell’Istria, 34137 Trieste, Italy; feras.kharrat@burlo.trieste.it (F.K.); nour.balasan@burlo.trieste.it (N.B.); erica.valencic@burlo.trieste.it (E.V.); ronald.rodriguesdemoura@burlo.trieste.it (R.d.M.); mariateresa.distazio@burlo.trieste.it (M.D.S.); barbara.borot@burlo.trieste.it (B.B.); alberto.tommasini@burlo.trieste.it (A.T.); adamopio.dadamo@burlo.trieste.it (A.P.d.); egidio.barbi@burlo.trieste.it (E.B.); domenicoleonardo.grasso@burlo.trieste.it (D.L.G.); 2 Department of Pharmacy, University of Salerno, Via Giovanni Paolo II 132, 84084 Salerno, Italy; valentina.golino@unina.it (V.G.); pcampiglia@unisa.it (P.C.); 3 Department of Medicine, Surgery and Health Sciences, University of Trieste, Strada di Fiume 447, 34149 Trieste, Italy; giulia.peri@studenti.units.it; 4 Department of Medical Laboratory Science, Hebron University, Hebron 90100, Palestine; qaisiyam@hebron.edu

**Keywords:** deep proteomics, tonsillar CD19+ cells, PFAPA, OSAS

## Abstract

Tonsils are secondary lymphoid organs that play a crucial role in the immunological response, with B cells being a major component involved in both innate and adaptive immunity. Periodic fever, aphthous stomatitis, pharyngitis, and adenitis (PFAPA) syndrome and obstructive sleep apnea syndrome (OSAS) are both common pediatric conditions involving tonsillar pathology. In both syndromes, the molecular pathways dysregulated in tonsillar B cells are still to be understood. The study aimed to unravel and compare the proteomic profiles of tonsillar CD19+ B cells isolated from pediatric patients with PFAPA (n = 6) and OSAS (n = 6) to identify disease-specific molecular signatures. B cells were isolated from the tonsillar tissue using magnetic microbeads (with a purity of 93.50%). Proteomic analysis was performed by nanoLC-MS/MS with both data-dependent (DDA) and data-independent acquisition (DIA) methods, followed by comprehensive bioinformatic analysis. By merging DDA and DIA datasets, a total of 18.078 unique proteins were identified. Differential expression analysis revealed 83 proteins increased and 49 proteins decreased in OSAS B cells compared to PFAPA B cells (fold change ≥ 1.5 or ≤0.6, *p* < 0.05). Distinct pathway enrichments were highlighted, including alterations in the regulation of PTEN gene transcription, circadian gene expression, inflammasome pathways (IPAF and AIM2), and the metabolism of angiotensinogen to angiotensin. Specific proteins such as p53, Hdac3, RPTOR, MED1, Caspase-1, Cathepsin D, Chymase, and TLR2 (validated by WB) were shown to be differentially expressed. These findings reveal distinct proteomic signatures in tonsillar B cells from patients with PFAPA and OSAS, offering novel insights into the pathophysiology and potential avenues for biomarker discovery.

## 1. Introduction

Representing the first line of defense against pathogens that enter the throat or nose, tonsils are secondary lymphoid organs composed of a combination of endothelial cells, immune cells, including B cells, T cells, macrophages, and other types of cells [1]. Tonsils change in size with age, being largest during childhood and decreasing in size in adults [2,3], and show changes in cellular composition [4]. B cells are the major cellular type in the tonsil tissue. They act in both innate and adaptive immunity [5]. Defective B-cell development is the main clinical manifestation of Bruton’s syndrome, where the tonsils are less developed or absent [6,7].

Periodic fever, aphthous stomatitis, pharyngitis, and adenitis (PFAPA) syndrome is an inflammatory disorder of childhood classically characterized by recurrent fevers, pharyngitis, stomatitis, cervical adenitis, and leukocytosis. Several studies have highlighted the roles of different cells in tonsil tissue, including IL-10-secreting B cells [8] and T cells in tonsillitis [9]. However, at the tissue level, there is a lack of knowledge behind the pathways involved in the outputs of PFAPA.

Memory B cells were reported to be increased in tonsils from patients with PFAPA [10] and expressed high levels of survival markers, such as BAFF-R and TACI, which suggests an immune dysregulation and a hyperactive microenvironment even during periods without fever. Besides the alteration in the B cell population, elevated levels of CD8+ T cells were reported, accompanied by high expression levels of *CXCL9*, *CXCL10*, and *CCL19* genes [11], which is one of the signs of persistent inflammation. Inflammation is a part of the immune system’s response to infections. In PFAPA, inflammation is triggered by an abnormal activation of the immune system, resulting in fever and other symptoms. The increased level of IL-1β and the activation of monocytes, neutrophils, and the components are all consequences of the inflammasome activation that occurs in PFAPA [12,13]. The periodic nature of this disease is one of its hallmarks that is not yet fully understood. A recent study suggested that B-cell adapter protein (*PIK3AP1*) and spondin-2 (*SPON2*) might play a role in the etiology of PFAPA by altering the DNA methylation [14].

Obstructive sleep apnea syndrome (OSAS) is characterized by recurrent episodes of reduced or absent breathing during sleep resulting from partial or complete obstruction in the upper airway. Disturbance in blood oxygenation typically occurs in patients with OSAS, leading to further complications that affect the quality of life [15]. In children, OSAS is caused by obstructive tonsils and adenoids and can be treated surgically by tonsillectomy or adenoidectomy. Tonsils from children with OSAS were reported to exhibit higher proliferative rates and increased levels of inflammatory cytokines [16] compared with those from children with recurrent tonsillitis (RT). B cells typically become active in the germinal centers of the tonsils, where they undergo somatic mutation and differentiate into either memory B cells or plasma cells. In OSAS, these germinal centers become larger, with a higher concentration of B cells within them [17]. A very recent study reported fewer naïve B cells and regulatory CD4^+^ T cells, and more plasma B cells compared with the enlarged adenoids [18]. Obstructive sleep apnea (OSA) can lead to metabolic dysfunction in the tonsil tissue, resulting from the intermittent hypoxia, which alters the insulin sensitivity and inflammation signaling [19]. Carrasco et al. reported an increase in B naïve population in the tonsils of severe patients with OSAS, but without an increase in Ki-67 expression, suggesting that defective differentiation and/or migration, rather than cellular proliferation, are the mechanisms behind this increase in B naïve population [20]. Several studies have reported that OSAS and circadian clock disruption may be associated with similar pathways. The interaction between hypoxia-inducible factor 1 (HIF-1) and rhythm clock components, such as PER, CLOCK, and BMAL1, suggests that hypoxia in Patients with OSAS can disrupt circadian rhythm clock regulation and metabolic dysregulation of glucose, lipids, and bile acid secretion [21,22].

Currently, there is little knowledge about the tonsillar proteome associated with PFAPA and OSAS. Proteomics methods were recently used to identify biomarkers for PFAPA in whole tonsil tissue. In a study by Mutlu et al. [23], an nHPLC LC-MS/MS system was employed for protein identification and label-free quantification, revealing differentially expressed proteins involved in the mitochondrial electron transport chain and ATP biosynthesis processes. However, there is still a need to furtherly investigate the dysregulated pathways behind PFAPA and OSAS and eventually identify biomarkers for these diseases. Hence, in our presented study, we aimed to study the proteomic profile of tonsillar B cells, which are the most abundant in tonsil tissue, from pediatric patients with PFAPA and OSAS. To our knowledge, this is the first study to investigate this specific cellular population from tonsil tissue.

## 2. Results

- **Enrolled population:**

The PFAPA group included three female and three male pediatric patients who underwent adenotonsillectomy. Their ages ranged from 3 to 9 years (median age 6 years). These patients had an early onset of the syndrome, with an average frequency of febrile episodes of approximately one per month. All were asymptomatic during afebrile intervals.

The OSAS group included one female and five male pediatric patients who also underwent adenotonsillectomy. Their ages ranged from 2 to 11 years. According to the **Brodsky Scale**, all children had tonsillar grades between **III and IV**, and were classified as **ASA physical status I–III**. These children experienced obstructive sleep apnea episodes lasting more than 10 s, occurring multiple times during the night.

- **Flow cytometry:**

**Figure 1:**
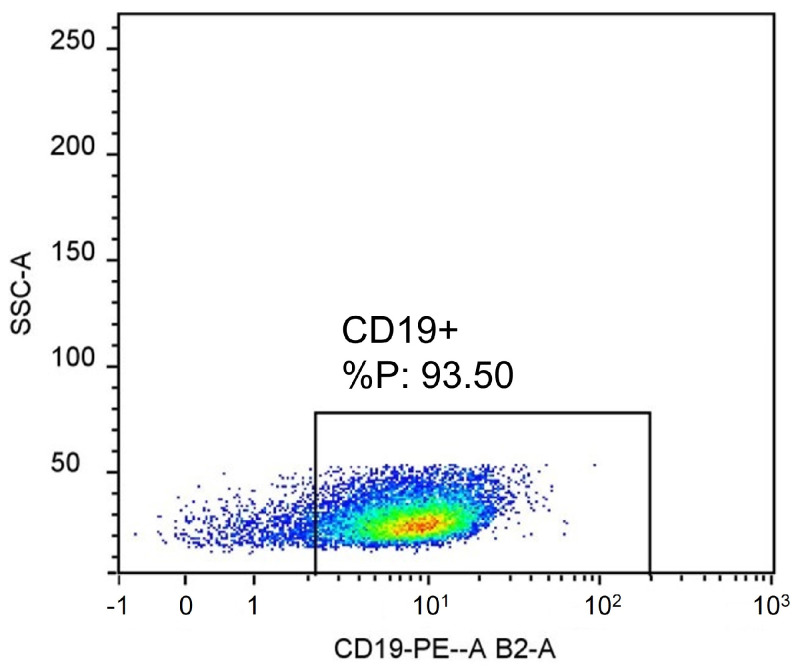
Cells isolated by CD19+ microbeads were positive for CD19+ antibody.

Flow cytometry results indicate that CD19+ cells can be isolated with a high purification rate from tonsil tissue, with 93.50% of the purified cells being positive for the CD19+ antibody (Figure 1).

- **Proteomics study:**

For deep proteomics analysis, we performed DDA and DIA. For the DDA data elaboration, we used Proteom Discoverer 3.1. As an initial step, we analyzed the DDA file using different search engines, including Amanda, Sequest, and Inferis. We identified 11,063 proteins. (Supplementary Materials File S1) with q-value < 0.05 and FDR < 1%.

The second step was processing Dia files. We used Spectronaut 19 software to process DIA files and identified 10,458 proteins with q < 0.01 and FDR < 1% (Supplementary Materials File S1).

**Figure 2:**
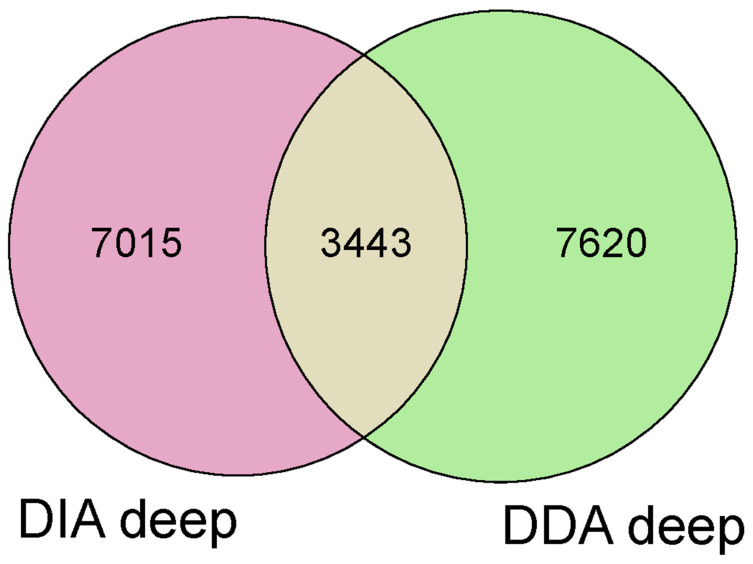
Intersection of DDA deep with DIA deep by Venn diagram for full proteomics data integration. DIA—data-independent acquisition, DDA—data-dependent acquisition.

The third step involved merging data from the two acquisitions (DIA+DDA), resulting in the identification of 18,078 unique proteins. Figure 2. The Mann–Whitney sum-rank test (*p* < 0.05) and the fold change ≥ 1.5 and ≤0.6 were applied to the 18,078 previously identified proteins. This analysis resulted in an increase of 83 proteins in OSAS with a fold change ≥ 1.5 and 49 proteins decreased in OSAS with a fold change ≤ 0.6 (Supplementary Materials File S1).

- **Bioinformatic Analysis**

In the next step, we performed a bioinformatic analysis using the Cytoscape software with the ClueGO plugin to identify differences in the proteomic profiles of tonsillar B cells between patients with PFAPA and those with obstructive sleep apnea. The bioinformatics tool was used for protein characterization based on their molecular function, biological processes, and pathways. The HUB genes network was also created to further understand the gene networks involved in the biology of B cells in PFAPA and OSAS.

**Figure 3:**
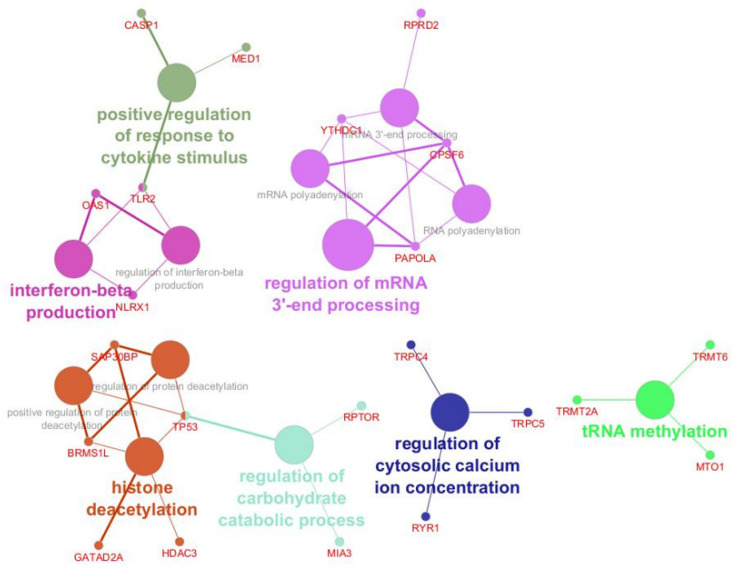
Biological processes analysis of the proteomic profile of tonsillar B cells revealed the proteins involved in the pathology of PFAPA and OSAS.

In terms of biological processes, the proteins were ranked by the regulation of mRNA 3’-end processing, regulation of cytosolic calcium ion concentration, tRNA methylation, histone deacetylation, interferon-beta production, and positive regulation of response to cytokine stimulus (Figure 3).

**Figure 4:**
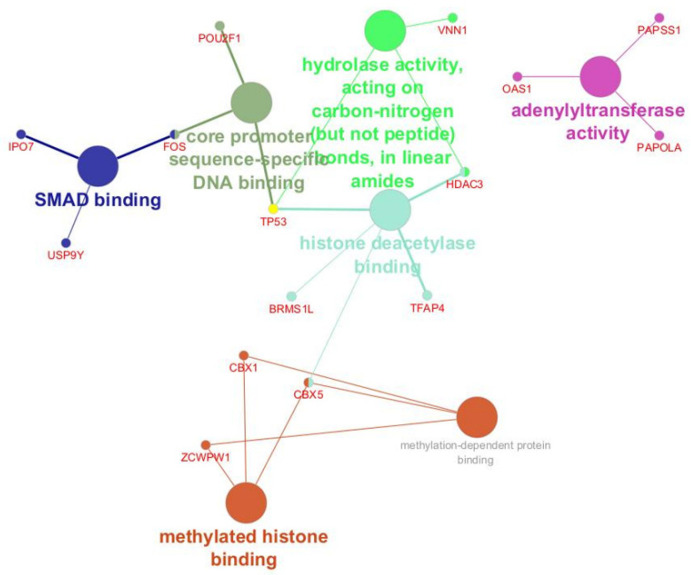
Molecular function analysis of the proteomic profile of tonsillar B cells show the functions of the proteins involved in the pathogenesis of PFAPA and OSAS.

Regarding the molecular function, proteins were ranked into Hydrolase activity, adenyltransferase activity, methylated histone binding, SMAD binding, and core promoter sequence-specific DNA binding (Figure 4).

**Figure 5:**
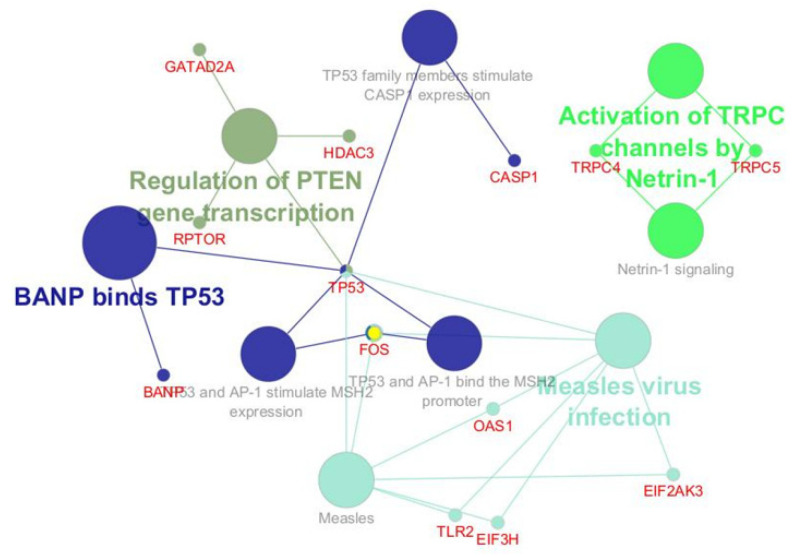
The pathways tool, revealed by analyzing the proteomic profile of tonsillar B cells, identified the pathways most involved in the pathogenesis of PFAPA and OSAS.

Analyzing the pathways revealed that the proteins were ranked by Regulation of *PTEN* gene transcription, activation of TRPC channels by Netrin-1, *binding of BANP to TP53,* and measles virus infection (Figure 5).

**Figure 6:**
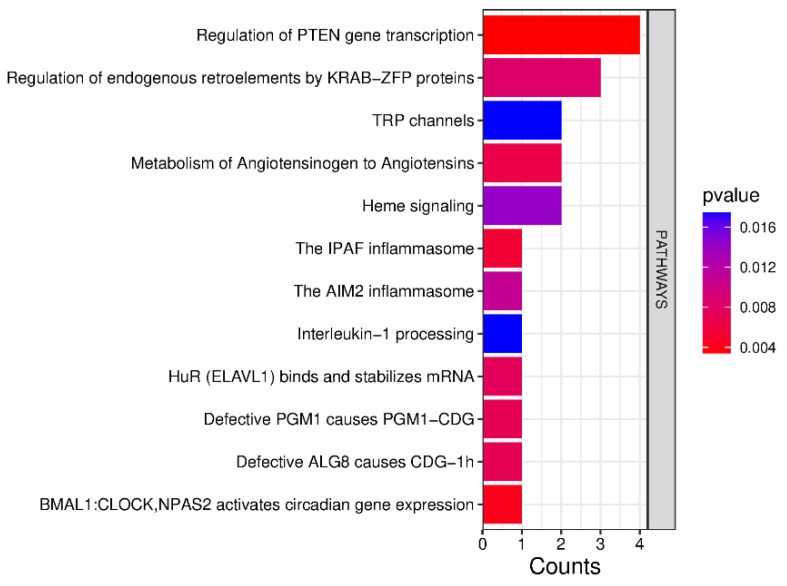
Reactome analysis revealed the regulation of *PTEN* gene transcription as the most differentially altered pathway in tonsillar B cells between PFAPA and OSAS.

The Reactome tool for the pathway database indicates that these proteins are primarily involved in the regulation of *PTEN* gene transcription, the regulation of endogenous retroelements by KRAB-ZFP proteins, TRP channels, the metabolism of angiotensinogen to angiotensins, the IPAF and AIM2 inflammasomes, and Interleukin-1 processing (Figure 6 and Table 1).

**Figure 7:**
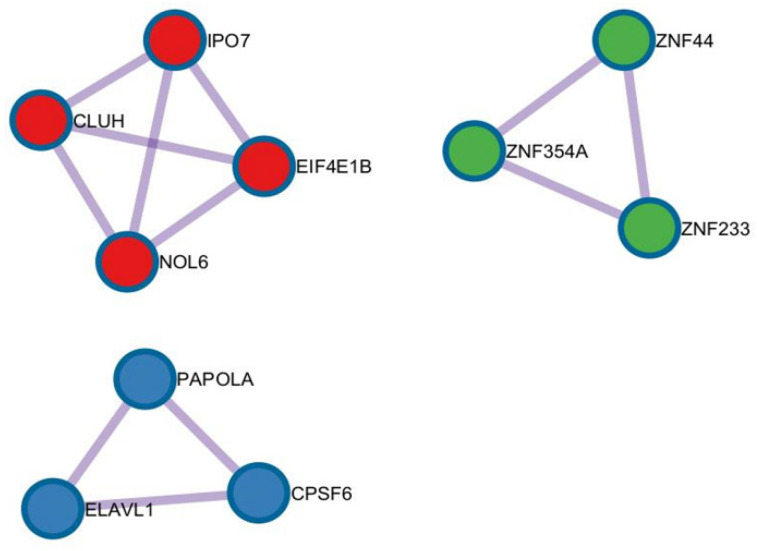
Hub genes analysis revealed the network of genes expressed in tonsillar B cells associated with the pathology of PFAPA and OSAS.

The hub genes analysis revealed three different clusters. The first cluster, which included green nodes, comprised three different genes from the zinc-finger protein family: *ZNF354A*, *ZNF44*, and *ZNF233*. The second cluster with red nodes included four different genes: *IPO7*, *CLUH*, *EIF4E1B,* and *NOL6*. The last cluster, which included blue nodes, consisted of *PAPOLA*, *ELAVL1,* and *CPSF6* (Figure 7).

- **Western blotting**

**Figure 8:**
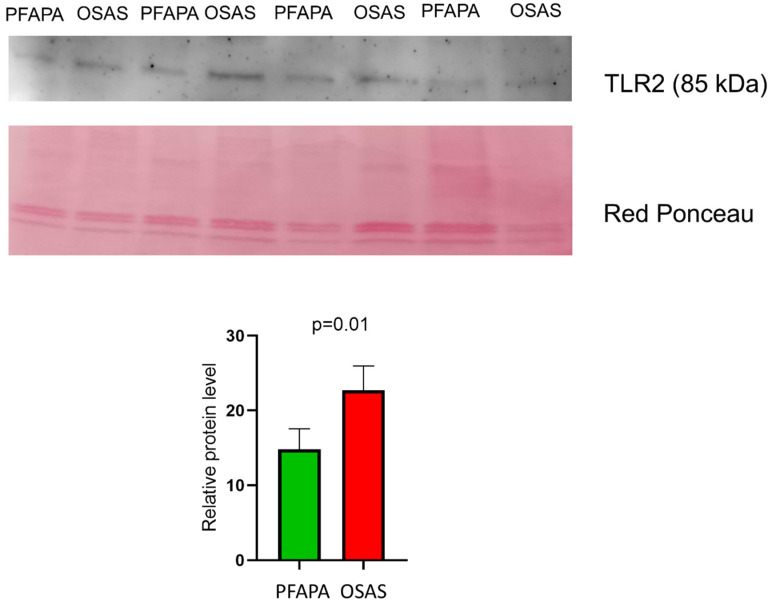
Western blotting of TLR2 from tonsillar B cells in PFAPA and OSAS. TLR2 is upregulated in B cells of patients with OSAS compared to patients with PFAPA (*p* = 0.01). The intensity of the immunostained bands was normalized to the total protein intensity, as measured from the same blot stained with Red Ponceau. The results are presented as a histogram, with each bar representing the mean ± standard deviation.

Toll-like receptor 2 was shown to be differentially expressed by the proteomics analysis performed on the CD19+ cells isolated from patients with OSAS compared to those affected by PFAPA. Western blotting analysis was performed to verify this finding (Figure 8).

## 3. Discussion

This study addressed different pathways in OSAS and PFAPA. In our opinion, the most significant finding concerns the regulation of the PTEN gene transcription pathway, which is among the primary drivers of cellular proliferation. Within the *PTEN* pathway, distinct protein expression patterns were observed between patients with OSAS and those with PFAPA. Specifically, the p53, histone deacetylase 3 (Hdac3), and transcriptional repressor p66-alpha (GATAD2 p66-alpha) proteins were upregulated in patients with OSAS compared to their levels in the other group, while the RPTOR protein from the same pathway was upregulated in PFAPA compared to patients with OSAS. p53 activation may be explained as a cellular response to the augmented hypoxic conditions and DNA damage associated with OSAS [24,25]. Concomitant increase in Hdac3 and *GATAD2 (p66-alpha)* can lead to increased *PTEN* activation and accumulation in the nuclei of cells. The *Hdac3* inhibitor can induce *PTEN* activation through acetylation [26]. *GATAD2* acts as a co-activator of P53 by enhancing *PTEN* nuclear translocation, which can be a factor in cellular processes [27]. In bacterial infections, the RPTOR protein is often upregulated, activating the mTORC1 pathway. This activation plays a role in the innate and adaptive immune responses by influencing lymphocyte expansion and cytotoxic responses. A study has shown that blocking Raptor/mTORC1 in the rockfish experiment leads to impaired immune responses and increased vulnerability to bacterial infection [28].

The results also indicated differential expression in the pathway, where BMAL1, CLOCK, and NPAS2 activate circadian gene expression. In this pathway, Mediator of RNA polymerase II transcription subunit 1 (*MED1*) was observed to be upregulated in OSAS compared with PFAPA. This increase may reflect a response to the inflammatory environment that potentially affects circadian rhythm disruption and immune gene expression [29]. Inflammasome pathways, including IPAF and AIM2, were also found to be differentially expressed between OSAS and PFAPA. The inflammasomes activate the Caspase-1 enzyme, which subsequently cleaves the inactive pro-IL-1β into its active form, which is then secreted to trigger inflammatory responses [30]. In addition, caspase-1 activation also leads to pyroptosis, a form of inflammatory cell death induced by bacterial pathogens [31].

In our study, the pathway of metabolism of Angiotensinogen to Angiotensins has also been shown to be differentially expressed between OSAS and tonsillitis. In this pathway, the Cathepsin D level was increased in CD19+ cells of patients with OSAS compared to those of patients with tonsillitis. This can be explained by the hypoxic conditions of OSASA, which might increase Cathepsin D expression to support tissue renin-angiotensin system (RAS) activation. Cathepsine D helps start the RAS cascade under stress by producing Angiotensin I when renin is absent or low [32]. Chymase protein was also shown to be upregulated in patients with tonsillitis compared to those with OSAS. Chymase is known to help amplify Angiotensin II levels locally, especially during chronic inflammation and infection [33]. In our patient’s case, Chymase is high in PFAPA, suggesting a similar role of its increased level, as shown upon infection, in mast cells. Mast cells release chymase, which generates Ang II, promoting inflammation [34].

Phosphoglucomutase-1 (PGM1) expression is upregulated in B cells of patients with PFAPA, which may be due to bacterial infections that lead to sustained B cell activation, requiring enhanced glucose metabolism and glycogen turnover, thereby upregulating PGM1 expression [35].

The Dolichyl pyrophosphate Glc1Man9GlcNAc2 alpha-1,3-glucosyltransferase protein, encoded by the ALG8 gene, was shown to be upregulated in B cells isolated from patients with OSAS compared to those with PFAPA. This protein adds the second glucose moiety to the lipid-linked oligosaccharide precursor (LLO, aka N-glycan precursor) required for subsequent N-glycosylation of protein, which is a critical process for proper protein folding in the endoplasmic reticulum [36,37]. This increase could reflect a response to hypoxia-induced stress and increase metabolic requirements associated with hypoxia-induced inflammation.

ELAV-like protein 1, also known as *HuR*, is an RNA-binding protein that stabilizes mRNAs containing AU-rich elements and regulates the expression of genes involved in cell survival, proliferation, and immune response [38]. Post-transcriptional regulation of mRNA by the RNA-binding protein *HuR* is crucial in B cells for the germinal center reaction and class-switched antibody production in response to thymus-independent antigens [39]. We observed an increased expression of HuR protein in B cells of patients with OSAS that could enhance mRNA stability and support cellular adaptation to low oxygen (hypoxic) conditions.

Zinc finger protein 354A (*ZNF354A*) is part of the Krüppel-associated box domain zinc finger proteins (*KRAB-ZFPs*) family, which are transcriptional repressors involved in maintaining genomic stability by silencing transposable elements and regulating endogenous retroelements [40]. Increased protein expression in B cells of our patients with OSAS may be due to stress response. Cells can upregulate KRAB-ZFPs, such as ZNF354, to maintain genomic integrity and regulate stress-responsive genes. Within the regulation of endogenous retroelements by the *KRAB-ZFP* proteins pathway, we observed differential expression of three key proteins between patients with OSAS and PFAPA in our study on B-cell proteomics. Particularly, *ZNF354* and *GATAD2A* (p66-α) are increased in patients with OSAS, while Chromobox homolog 5 (CBX5) is upregulated in the PFAPA group. This variation is likely related to the environmental pressures of each disease. OSAS is characterized by hypoxia that leads to oxidative stress and inflammation. It likely promotes a dynamic repression response [41], including KRAB zinc finger proteins and their corepressors, such as *GATAD2A*, which maintain genome stability under fluctuating oxygen levels [42]. PFAPA is characterized by persistent antigenic stimulation that may require a more stable form of epigenetic repression. The increased CBX5 in this group of patients may support this model, as CBX5 binds to H3K9me3-marked chromatin to maintain the long-term silencing of retroelements [43,44].

The Heme signaling pathway involves various cellular processes, such as heme production, cell survival, and erythropoiesis. Different factors can regulate the Heme pathway, including transcriptional factors, signaling molecules, and epigenetic regulators [45]. *MED1* and *Hdac3* proteins were shown to be upregulated in our study in patients with OSAS. At the same time, the direct interaction between both proteins within the Heme pathway is not clearly reported in the literature. Probably, they indirectly influence the pathway through their roles in gene expression regulation.

Short transient receptor potential channels 4 (TRPC4) and 5 (TRPC5): We observed increased expression of Short Transient Receptor Potential Channels 4 and 5 in CD19+ B cells from patients with OSAS. A study has shown that TRPC5 promotes the response to intermittent hypoxia-induced cardiomyocyte injury through oxidative stress [46]. Another study found that the TRPC5 channel is involved in the cardiac damage related to OSAS in rats [47]. Similarly, TRPC4 expression increases under hypoxic conditions, leading to enhanced calcium influx and the activation of transcription factors like AP-1 [48]. The upregulation of TRPC4 and TRPC5 channels in B cells may reflect an adaptive response to the hypoxic environment.

When using molecular function ontology, most of the proteins exhibit protein binding activity (methylated histone binding, histone deacetylase binding, core promoter binding, and SMAD binding), highlighting the critical key role of these proteins in immune tolerance and inflammation in PFAPA. At the same time, other proteins take part in hydrolase activity and adenylyltransferase activity.

The B cell proteomics of PFAPA identified TP53, HDAC3, TFAP4, and BRMS1L as functionally significant proteins involved in histone modification and interconnected with methylated binding proteins (at CBX5), core promoter binding protein (at TP53), and hydrolase activity proteins (*HDAC3*). The results indicate their involvement in the pathology of PFAPA. *TP53* is involved in regulating the abnormal proliferation or apoptosis of B cells caused by chronic infection and inflammation [49]. HDAC3 perturbation is likely to alter gene expression patterns in B cells, leading to a lack of immune tolerance and thereby enhancing susceptibility to recurrent infections [50,51]. *TFAP4* is involved in regulating the cell cycle. Its role in PFAPA is not well-investigated, and its dysregulation might trigger abnormal B cell proliferation and subsequent tissue damage [52]. *BRMS1L* is involved in chromatin remodeling, suggesting that it could affect the transcription of several target genes in B cells [53]. Dysregulation of *BRMS1L* can lead to uncontrolled B-cell activation and impaired immune responses.

*OAS1*, *PAPSS1*, and *PAPOLA* were identified as functionally significant proteins in B-cells of PFAPA. OAS1 is an interferon-stimulated gene (ISG) that plays a key role in the antiviral response, and its dysregulation can lead to chronic inflammation [54]. *PAPSS1* is required for the sulfation of carbohydrates, lipids, and proteins. It plays a crucial role in both cellular metabolism and signaling [55]. The disruption in the sulfation process causes metabolic changes in B cells during chronic inflammation that can affect cytokine signaling, antigen presentation, or immune cell interactions [56,57]. *PAPOLA* is involved in mRNA polyadenylation, which regulates mRNA stability, translation efficiency, and gene expression control of immune cells. This activity influences cytokine production and immune stimulation [58,59]. *PAPOLA* overexpression could indicate increased transcription activity of B cells, with possible elevated synthesis of inflammatory mediators. *PAPOLA* overactivity could lead to excessive immune stimulation and tissue damage in PFAPA.

For biological processes, most proteins were involved in epigenetic regulation through histone deacetylation, mRNA processing at the 3’ end, tRNA methylation, interferon beta production, and cytokine stimulus regulation. Other major processes included the regulation of carbohydrate metabolism and the regulation of intracellular calcium levels. The results demonstrated the importance of epigenetic control and modulating the gene expression patterns in B cell development by regulating the histone deacetylation activity [60]. Protein plays a crucial role in tRNA methylation, regulating both protein synthesis and protein quantity. *MTO1* facilitates proper mitochondrial protein synthesis and cellular energy metabolism [61], which are crucial for B cell function and viability [62]. Its dysregulation may result in mitochondrial dysfunction, metabolic adaptation of B cells, and defective immune responses (suppressing antibody production). The tRNA methyltransferases (*TRMT6* and *TRMT2A*) control translation efficiency and RNA stability under stress responses [63,64]. Their abnormal function may cause translation errors and defective antibody production under chronic inflammation of PFAPA. Moreover, the cluster of proteins involved in 3’ mRNA processing has a significant role in determining the quality and quantity of the targeted mRNA, which is critical for B cell development [65,66].

The Toll-like receptor 2 (TLR2) pathway is crucial for B cell activation and innate immunity following bacterial and viral infections [67,68]. Chronic inflammation correlated with PFAPA may lead to dysregulated TLR2 signaling in B cells, affecting the immune response. *NLRX1* [69], *MED1*, and *CASP1* [70] are proteins that bind and modulate *TLR2* activity. Their dysregulation may lead to uncontrolled TLR signaling, excessive production of pro-inflammatory cytokines, and impaired antibody production.

The regulation of the carbohydrate catabolic process is crucial for B-cell metabolism and immune function (antibody glycosylation and presentation) [71]. In PFAPA, chronic immune activation requires metabolic adaptations to sustain B-cell function in an inflammatory environment. The proteins *RPTOR*, *MIA3*, and *TP53* affect the energy state of B cells [72,73]. B cells use mainly glycolysis and oxidative phosphorylation to meet their energy demands during activation and antibody production. In chronic inflammation, B cells shift towards aerobic glycolysis, increasing glucose uptake to sustain immune responses. Thus, dysregulation in carbohydrate metabolism can lead to the hyperactivation of B cells, promoting chronic immune responses in PFAPA. *mTORC1*, controlled by *RPTOR*, induces glycolysis and glucose uptake for the proliferation of B cells and antibody secretion [74]. *MIA3* influences ER function and glucose metabolism and is involved in protein secretion, particularly cytokine and antibody secretion [75]. *TP53* inhibits exaggerated glycolysis [76], preventing uncontrolled B cell proliferation. *TP53* malfunction results in uncontrolled glycolysis, which supplies energy for chronic B cell activation and reduces apoptosis, leading to inflammation. Furthermore, *TP53* is connected to histone modification, indicating a crosstalk between cellular metabolism and epigenetic control [77].

A cluster of proteins is involved in regulating calcium signaling. Upon activation of B cell receptors (BCRs), the entry of calcium plays a central role in stimulating downstream signaling pathways, as well as controlling B cell proliferation, differentiation, and cytokine secretion [78,79]. *TRPC4*/*TRPC5* upregulation can lead to enhanced calcium influx and hyperactivation of B cell responses (overproduction of antibodies and chronic inflammation). *TRPC4*/*TRPC5* downregulation can lead to impaired B cell activation and an antibody production defect [80]. *RYR* is involved in ER calcium release, and its dysregulation leads to defective calcium mobilization and impaired immune response [81].

Pathway enrichment analysis revealed the protein related to the *PTEN* pathway. The *PTEN* pathway is the classical regulator of B cell development, proliferation, activation, and tolerance through its negative regulation of *PI3K*/*AKT* signaling. Dysregulation of the *PTEN* pathway results in persistent activation of *the PI3K/AKT pathway, leading to B-cell survival and a* prolonged immune response. *PTEN* modulates germinal center development, in which B cells mature and secrete antibodies [82,83]. Dysregulation of *PTEN* expression might disrupt B cell differentiation, leading to aberrant or excess antibody responses in PFAPA. Besides its classical role, *PTEN* is interconnected with cellular energy status (via *mTOR*) [82], chromatin remodeling (via *HDAC3*) [51], and apoptosis (via *TP53*) [84], making it a leading regulator of B-cell maturation and development. The dysregulation of the *PTEN* pathway leads to the hyperactivation of B cells, chronic inflammation, and the overproduction of antibodies, which can exacerbate the chronic inflammation. If upregulated, it can suppress immune responses, rendering the host susceptible to bacterial or viral infections.

Transcriptional regulator protein (*BANP*) controls *TP53* activity [49] and chromatin remodeling [85] to suppress the uncontrolled B cell proliferation and regulate *NF-κB* activity [86], which is accountable for cytokine production and immune signaling. Dysregulation of *BANP* may lead to reduced apoptosis and increased NF-κB activity, resulting in elevated levels of inflammatory cytokines.

The identification of the circadian Rhythm genes is relevant. Proteins of the biological clock are essential for regulating B cells. Lymphocyte circulation, infiltration, recruitment, maturation, and cytokine release are also regulated by the clock genes [87,88]. B cell counts have a significant peak at night with lower levels during the daytime. Circadian rhythms in B cells modulate activation, differentiation, and antibody secretion in response to infection [89]. In PFAPA, the dysregulation of circadian proteins might account for abnormal immune responses and inflammation. *CLOCK* and *BMAL1* are essential transcription factors that regulate the expression of genes involved in the circadian rhythm [90]. These proteins dictate the timing of B cell activation. Their disruption can result in hyperactive B cells (altered timing of the immune response), defective immune responses (overproduction of cytokines), and aberrant B cell tolerance. The misalignment in circadian rhythm can also affect the ability of B cells to mount an effective immune response to pathogens, increasing the probability of recurrent infections in the tonsils.

These hub genes exhibit key regulators and a signature of hyperactivation of B cells and immune response in PFAPA. These include the specific *ZNF* proteins (*ZNF44*, *ZNF233,* and *ZNF354A*). Although their functions in B cells are not well-studied, their general role as transcriptional regulators suggests their involvement in regulating the immune gene expression of B cells. *ZNF* proteins may target the NF-κB Pathway (inflammation regulation), the JAK-STAT Signaling Pathway (cytokine response), the *TGF-β* Pathway (immune tolerance regulation), and epigenetic regulation (chromatin structure and long-term gene expression modification in B cells). In PFAPA, their dysregulation can be a signature of B-cell activation.

Mediators of protein translation (*NOL6* and *EILF4E1B*) were also identified as hub genes, highlighting the quality control on RNA and protein synthesis that could result in insufficiency of immune response. *IOP7*, a nuclear transport protein, is required for transcription factors to shuttle into the nucleus. When dysregulated, it may perturb normal signaling, affecting B cell differentiation and the formation of memory response, making the subject more susceptible to recurring infection. *CLUH* is encoded for mitochondrial regulation and metabolism. When *CLUH* is dysregulated, B cells may have an impaired energy metabolic state, which affects their ability to mount an effective immune response and clear pathogens.

Three hub genes were filtered and primarily involved in mRNA polyadenylation and stability (*PAPOLA*), mRNA processing (*CPSF6*), and mRNA stability for inflammatory cytokines (*ELAVL1*). The three genes are crucial to B cell function and immune regulation. Being dysregulated in PFAPA, they can cause aberrant immune activation and defective antibody production. These 10 hub genes can be further investigated as potential biomarkers of PFAPA and therapeutic targets.

The main limitation of this study is the obvious lack of a comparison with tonsils from age-matched healthy subjects. The availability of biological samples from healthy tonsils is extremely limited, largely due to clear ethical considerations. Another limit is the limited sample size. No other studies were available suggesting a possible number of needed specimens. However, the high-throughput nature of the proteomic methodology allowed us to obtain statistically significant results. Moreover, the availability of tonsil specimens of children undergoing tonsillectomy for PFAPA is overall limited. A further limitation is that, for technical verification, the vast majority of protein extracts obtained from purified cells were used for spectrometric analysis, making it impossible to conduct further experiments for verification. Finally, regarding the in situ immunohistochemistry, we were not able to prepare the paraffin-embedded block because all the tonsillar tissue was used for CD19+ purification.

However, the ability to compare two conditions primarily affecting tonsillar biology allowed for the acquisition of important differential data that can form the basis of future studies aimed at validating biomarkers and identifying individualized therapeutic targets.

## 4. Materials and Methods

### 4.1. Patient Enrolment

Six patients diagnosed with PFAPA and six patients with OSAS were enrolled at the Institute for Maternal and Child Health—IRCCS “Burlo Garofolo” (Trieste, Italy) during 2024. The Institutional Research Board approved this study, and all the parents or legal representatives signed the informed consent form.

**Inclusion criteria for PFAPA:**

- Regularly occurring fevers (without evidence of upper respiratory infection).
- Onset prior to age 5 years.
- At least one of the following is associated with fevers: aphthous stomatitis, pharyngitis, cervical adenitis.
- Resolution of symptoms with a single, low dose of Betamethasone (0.1 mg/kg po once in the first 24 h of a febrile episode).
- Normal growth as assessed by World Health Organization growth charts.
- Asymptomatic between episodes.

**Inclusion criteria for OSAS:**

- Age (2–11).

Symptoms of OSAS (apnea, heavy snoring, sleep disturbance confirmed by video recording).

- Tonsil hypertrophy 3 or 4 according to the Brodsky scale [91].

**Exclusion criteria for PFAPA:**

- Genetically defined autoinflammatory disorders (familial Mediterranean fever, Hyper-IgD syndrome/mevalonic kinase deficiency, TNF receptor-associated periodic syndrome, cryopyrin-associated periodic syndrome).
- Cyclic neutropenia.
- Immunodeficiency or autoimmunity.

**Exclusion criteria for OSAS:**

- Craniofacial abnormality.
- Neuromuscular disease.
- Chromosomal abnormality.
- Previous adenotonsillar surgery.
- Bleeding disorder.
- Cardiopulmonary disease.
- History of recurrent tonsillitis.

### 4.2. Sample Collection

Tonsils were obtained from the surgical unit and kept in physiological solution for less than three hours at 4 °C before processing.

### 4.3. Isolation of Mononuclear Cells (MNCs) from Human Palatine Tonsils

MNCs were isolated as reported by Assadian et al. [92]. Briefly, the tonsil was immersed in a precooled solution of sterile Hanks balanced salt solution (HBSS) supplemented with 5% fetal bovine serum (FBS) (ECS0170L, Euroclone S.p.A., Pero, Italy), 10 mM Glutamine (ECB3000D, Euroclone S.p.A., Pero, Italy), 0.05 mg/mL Gentamicin/ Amphotericin B (Gibco, R-015-10; Thermo Fisher Scientific, Waltham, MA, USA), and 1% Antibiotic mix (Penicillin/Streptomycin (ECB3001D, Euroclone S.p.A., Pero, Italy)). The tonsil was cut into small fragments and squeezed through a 100 µm strainer using the plunger end of a plastic syringe. The resulting cellular suspension, in a volume of 15 mL, was then transferred into a 50 mL Falcon tube containing 10 mL of the gradient solution, Lympholyte (DVCL5015R, Cedarlane Lab, Burlington, ON, Canada), and centrifuged at 700× *g* for 40 min without brake. At the end of the centrifugation, MNCs were visible as a fluffy white layer at the interface and collected carefully. The cells were frozen in −80 °C in freezing medium composed of 90% FBS and 10% DMSO, Dimethyl sulfoxide (EMR031100, Euroclone S.p.A., Pero, Italy)

### 4.4. Isolating CD19+ Cells with the Magnetic Microbeads

Cells were thawed and centrifuged at 300× *g* at 4 °C. Then, the freezing medium was discarded, and the cells were resuspended in cold PBS and counted by Denovix Celldrop BF (DeNovix Inc., Wilmington, DE, USA) with trypan blue.

CD19+ cells were isolated using Miltenyi magnetic microbeads (130-050-301) using the LS columns (130-042-401) according to the manufacturer’s instructions.

### 4.5. Protein Extraction

Upon being captured by the magnetic beads, the cells were centrifuged, and SDS lysis buffer (Thermo Scientific A40006) was added to the pellet, which was then heated at 95 °C for 5 min. The samples were left to cool to room temperature, then subjected to ultrasonication for 10 seconds, followed by centrifugation at 12,000 × g for 15 minutes at 4 °C. The supernatant containing the protein extract was collected and kept at −80 °C. The protein concentration was determined using the Bradford assay.

### 4.6. Flow Cytometry

One sample was analyzed by the flow cytometry Miltenyi MACSQuant (Miltenyi Biotech, Germany) to verify the protocol. In brief, 100,000 cells from each of the following populations—isolated CD19+ cells, flowthrough cells, and unpurified cells—were labeled with both the CD19+ antibody anti-human, PE, REAfinity™, Clone REA675 (Miltenyi 130-114-172) and the related control antibody (Miltenyi 130-113-450).

### 4.7. Proteomics

An amount of 20 µg of protein extract was digested with the EasyPep™ MS Sample Prep Kits (Thermo Fisher, Waltham, Massachusetts, United States), as previously described [93]. Analysis was performed by using nanoflow ultra-high performance liquid chromatography-high-resolution MS using an Ultimate 3000 nanoLC (Thermo Fisher Scientific, Bremen, Germany) coupled to an Orbitrap Lumos tribrid mass spectrometer (Thermo Fisher Scientific, Waltham, Massachusetts, United States) with a nanoelectrospray ion source (Thermo Fisher Scientific, Waltham, Massachusetts, United States), as previously described [94]. One μL of the digest was loaded and trapped on a PepMap trap column for 1.00 min at a flow rate of 40 μL/min (Thermo Fisher, Waltham, Massachusetts, United States), and then peptides were separated by a C18 reversed-phase column (250 mm × 75 μm I.D., 2.0 µm, 100 Å, EasySpray, Thermo Waltham, Massachusetts, United States). A linear 90 min gradient was performed. MS analysis was performed in DDA mode, with an MS1 range of 375–1500 *m*/*z* set at a resolution of 120,000, while MS2 was performed at a resolution of 15,000. The isolation window was set to 1.6 Da normalized, and a collision energy (HCD) value of 30 was applied.

For MS/MS, the maximum ion injection time for the MS/MS (OT) scan was set to 50 ms, and ACG values were set to standard. The dynamic exclusion was set to 30 s.

For DIA, a first MS scan was performed at a resolution of 120,000. DIA was performed in OT at 15,000 resolution. DIA was performed with 10 Dalton isolation windows, and the AGC target and maximum ion injection time were set to custom values of 200 ms and 40 ms, respectively. Loop control was set to N = 30. HCD was used with a collision energy value of 30.

Proteome Discoverer 3.1 was used for the DDA raw data analysis, which was performed using Sequest HT, AMANDA3.0, Chimerys, and INFERiS search engines. The following parameters were used: enzyme trypsin, maximum missed cleavages of 2, precursor mass tolerance of 10 ppm, and fragment mass tolerance of 0.02 Da. Carbamidomethylation was used as a fixed modification, while methionine oxidation was used as the variable. Proteins were considered identified with at least one unique peptide, setting a false discovery rate (FDR) threshold of <1%. The DIA raw data were analyzed using Spectronaut 19. The direct DIA (deep) tool (Spectronaut 19) was used for identification. Carbamidomethylation was used for fixed modification, while acetylation and oxidation were used for variable modifications. The FDR < 1% and proteins were considered to be identified with at least one unique peptide.

### 4.8. Bioinformatic Analysis

For bioinformatic analysis, the Reactome tool (https://reactome.org/PathwayBrowser/#TOOL=AT, accessed on 1 July 2025) was used for pathway identification. The Venn diagram and Enriched Horizontal Bars were generated using the SRplot online platform [95] (https://www.bioinformatics.com.cn/en, accessed on 1 July 2025), and were used for data correlation in proteomics and enriched bar plots. The biological processes, molecular functions, and Pathway identification were generated using Cytoscape software 3.10 with a significance level of *p* < 0.05.

### 4.9. Western Blotting

Western blotting was performed as described in [93]. Briefly, 15 ug of protein were loaded onto a 4–20% precast gel, then transferred to a nitrocellulose membrane. The membrane was blocked by treatment with 5% defatted milk in TBS-Tween 20 and incubated overnight at 4 °C with antibodies against TLR2 (Invitrogen, JM22-41) at a dilution of 1:500 (rabbit monoclonal). Nitrocellulose membrane was washed three times with TBS-Tween 0.05% and incubated with HRP-conjugated anti-rabbit IgG (1:3000). SuperSignal West Pico Chemiluminescent (Thermo Fisher Scientific Inc., Ottawa, ON, Canada) was used for protein band signal visualization. The intensities of the immunostained bands were normalized with the total protein intensities measured by staining the membranes from the same blot with a Red Ponceau solution (Sigma-Aldrich, St. Louis, MO, USA).

### 4.10. Statistical Analysis

Differences between patients and controls were considered significant when proteins showed a fold change ≥ 1.5 and ≤0.6 and satisfied the Mann–Whitney U test (*p* < 0.05). Analyses were conducted using the RStudio script (https://rstudio-education.github.io/hopr/starting.html, accessed on 1 July 2025).

## 5. Conclusions

This study provides the first comprehensive proteomic comparison of isolated CD19+ B cells from pediatric patients with PFAPA and OSAS, highlighting distinct molecular signatures of these conditions within the tonsillar microenvironment. Notably, the regulation of *PTEN* gene expression was dysregulated, with proteins such as p*53* and *Hdac3* upregulated in OSAS B cells and *RPTOR* upregulated in PFAPA B cells, suggesting alterations in cellular proliferation, survival, and metabolic control tailored to each disease condition. Additionally, the alterations in circadian rhythm-associated proteins, inflammasome components, and angiotensin metabolism suggest diverse immune and metabolic responses in B cells. The upregulation of *TLR2* in OSAS B cells, which was confirmed by Western blotting, also highlights the distinct innate immune activation. The proteomic differences collectively show how tonsillar B cells adapt to the conditions correlated with OSAS (such as hypoxia and metabolic stress) and PFAPA (which is characterized by recurrent inflammation involving infectious or autoinflammatory causes). The identified hub genes and dysregulated pathways, which are involved in epigenetic regulation, mRNA processing, calcium signaling, and carbohydrate metabolism, further highlight the complex B cell responses in these conditions.

This study suggests several mechanisms exerted by differential proteins and pathways, including cell proliferation, apoptosis, and the secretion of inflammatory factors. Future studies should target these mechanisms to provide evidence of the pathological mechanisms. Future research trends could also address the effects of hypoxia by utilizing in vitro models for the two disorders, as well as conducting correlation analysis between protein expression and clinical parameters (hypoxia index of OSAS, PFAPA attack frequency) in a larger population. Regarding the potential future applications of these findings in the real world, both diseases currently lack curative medicine, and surgery remains the gold standard option for the most severe cases. Having a drug to target these diseases may not only improve treatment and reduce symptoms of the milder forms but also reduce the need for surgery, limiting its related risks and costs.

## Figures and Tables

**Table 1:** List of proteins and their pathways identified to be differentially expressed in tonsillar B cells from patients with PFAPA and OSAS included in the study.

Pathway	Protein	UniProt Code	PFAPA	OSAS
**Regulation of PTEN gene transcription**	Cellular tumor antigen p53	P04637	-	Increase
	Histone deacetylase 3	O15379	-	Increase
	Transcriptional repressor p66-alpha	Q86YP4	-	Increase
	Regulatory-associated protein of mTOR	Q8N122	Increase	-
**BMAL1:CLOCK, NPAS2 activates circadian gene expression**	Mediator of RNA polymerase II transcription subunit 1	Q15648	-	Increase
**IPAF and AIM2 inflammasomes**	Caspase-1	P29466	Increase	-
**Metabolism of Angiotensinogen to Angiotensins**	Cathepsin D	P07339	-	Increase
	Chymase	P23946	Increase	-
**Defective PGM1 causes PGM1-CDG**	Phosphoglucomutase-1	P36871	Increase	
**Defective ALG8 causes CDG-1h**	Dolichyl pyrophosphate Glc1Man9GlcNAc2 alpha-1,3-glucosyltransferase	Q9BVK2	-	Increase
**HuR (ELAVL1) binds and stabilizes mRNA**	ELAV-like protein 1	Q15717	-	Increase
**Regulation of endogenous retroelements by KRAB-ZFP proteins**	Zinc finger protein 354A	O60765	-	Increase
	Transcriptional repressor p66-alpha	Q86YP4	-	Increase
	Chromobox protein homolog 5	P45973	Increase	-
**Heme signaling**	Mediator of RNA polymerase II transcription subunit 1	Q15648	-	Increase
	Histone deacetylase 3	O15379	-	Increase
**Interleukin-1 processing**	Caspase-1	P29466	Increase	-
**TRP channels**	Short transient receptor potential channel 4	Q9UBN4	-	Increase
	Short transient receptor potential channel 5	Q9UL62	-	Increase

## Data Availability

The data presented in this study are available on request from the corresponding author.

## Supplementary Materials

The following supporting information can be downloaded at: https://www.mdpi.com/article/10.3390/ijms26146621/s1.
